# Exploring Australian night shift workers’ food experiences within and outside of the workplace: a qualitative photovoice study

**DOI:** 10.1017/S1368980023001519

**Published:** 2023-11

**Authors:** Gloria KW Leung, Kate E Huggins, Maxine P Bonham, Sue Kleve

**Affiliations:** Department of Nutrition, Dietetics and Food, Monash University. Level 1, 264 Ferntree Gully Road, Notting Hill, VIC 3168, Australia

**Keywords:** Shift work, Qualitative, Workplace, Night work, Dietary pattern, Dietary intake

## Abstract

**Objective:**

Night shift workers are at a 20 to 40 % increased risk of metabolic diseases, which may be associated with their disrupted eating patterns. This qualitative study explores factors that influence night shift workers’ eating habits, within and outside of the workplace, to identify target areas for health promotion strategies.

**Participants and Setting:**

Eligible participants resided in Australia, working at least three overnight shifts per month.

**Design:**

The photovoice method was used, whereby participants were asked to take photos that represent their typical eating habits. These photos were incorporated as prompts in a semi-structured interview, which explored factors influencing eating habits on night shifts and days-off and perceptions and enablers to healthy eating.

**Results:**

Ten participants completed the study. Thematic analysis generated four main themes, which were mapped onto the Social Ecological Model (SE Model). Aligned with the SE Model, our results show night shift workers’ eating habits are influenced by intrapersonal, interpersonal and (work) organisational levels. Participants reported that at work, appropriate food preparation facilities are required to enable healthy food choices. Poor shift work rostering leads to prolonged fatigue on days-off, limiting their ability and motivation to engage in healthy eating. Consequently, night shift workers seem to require additional supports from their social networks and enhanced food literacy skills, in order to adopt/ maintain healthy eating behaviours.

**Conclusions:**

Night shift work creates individual and environmental barriers to healthy eating, which are present during and outside of work. Health promotion strategies for this population should include multiple approaches to address these barriers.

Epidemiological studies have shown that shift workers are at 20 to 40 % increased risks of metabolic diseases compared with those who exclusively work during the day (‘9 to 5’)^(1–4)^. A meta-analysis including twelve observational studies (226 652 participants) reported that the odds for shift workers (any type) in developing diabetes is 9 % higher than day workers; with those who work rotational shifts (combination of day, afternoon and night) experiencing 42 % increased odds^(2)^. A similar pattern is observed in a pooled analysis of twenty-one studies (320 002 participants) examining risks of ischaemic heart disease, with shift workers experiencing a 13 % increased risk and those engaging in permanent night shifts experiencing 44 % increased risk^(5)^. Within industrialised countries, approximately 20 % of employees engage in some form of shift work (e.g. permanent night, rotating or permanent day shift)^(6)^, accounting for 1·9 million individuals in Australia working across multiple industries such as healthcare, hospitality and transport^(7)^.

Due to their atypical working hours, night shift workers often adopt an irregular eating pattern, with frequent small meals throughout the 24-h day^(8–10)^. Such temporal eating pattern is misaligned with the regulation of our circadian clock system^(11)^, which programs us to eat during the day and fast during the night^(12)^. Snacking during night shift is especially common, characterised by discretionary snacks and beverages^(8,13)^. It has been speculated that the combination of eating the ‘wrong food’ and at the ‘wrong time’ is one of the causal factors to shift workers’ increased risks of metabolic diseases. Within Australia, there has been limited qualitative studies that explored the drivers behind such eating habits during night shifts. The majority of studies involved specific occupations^(13,14)^, reporting on factors that were unique to the job.

Cross-sectional studies have suggested that the difference in dietary intake between night and day workers extend beyond foods consumed during the night shift. Compared with permanent day workers, night shift workers have been reported to have a higher daily saturated fat intake^(15)^ and a lower daily vegetable intake^(16)^. This may be an indication that shift work leads to unhealthy eating habits outside of the night shift setting, exacerbating shift workers’ risk of metabolic diseases. Thus far, there has been little investigation on shift workers’ eating habits whilst not at work.

Despite night shift workers’ suboptimal diet quality, there is currently a lack of workplace dietary interventions, tailored at the prevention of metabolic diseases for this at-risk population. In order to develop practical and feasible dietary interventions, it is crucial to understand the drivers behind what, when and why night shift workers eat, both during and outside of work. Therefore, this qualitative study aimed to explore Australian night shift workers’ experience with food and eating, both within and outside of the workplace. The photovoice method was utilised in combination with semi-structured interview, which empowered participants to act as ‘knowledge owners’ and convey experiences that are meaningful to them through their captured photos^(17)^. This study will generate a deeper understanding into night shift workers’ overall food experiences, providing important points of considerations in the development of nutrition-related health promotion strategies for this population, which may take place within or beyond the workplace context.

## Methods

Recruitment for this qualitative study commenced in July 2019, and data collection was completed in January 2020. This study was conducted according to the guidelines laid down in the Declaration of Helsinki, and all procedures involving research study participants were approved by a University Human Research Ethics Committee (project ID: 19 509). The study is reported according to the Standards for Reporting Qualitative Research (SRQR) Guidelines (see online supplementary material, Appendix 1)^(18)^.

This research was grounded in pragmatism, which focuses on finding practical solutions to societal problems^(19)^. Through exploring Australian night shift workers’ food and eating experiences, this research contributed to the understanding and solutions to the nutrition problem identified, i.e. the increased risks of metabolic diseases observed in night shift workers.

The first author is professionally trained as a dietitian and has been engaged in shift work research for the past 6 years. Critical reflexivity was employed throughout the study, whereby the researcher acknowledged her background as a nutrition professional and reflected on how that would affect the decisions made during the research process and data interpretation^(20)^.

### Participants and sampling

Shift workers are known to be difficult to engage in research. The aim of participant sampling was therefore to achieve sufficient information power, rather than data saturation^(21)^. To achieve this, a purposive sampling strategy was utilised, aiming for maximum variation in gender, type of shift schedule and occupation. This allowed us to capture unique opinions and recounts of experience from our target group, maximising data richness. Eligible participants were night shift workers above 18 years of age, who had at least 6 months of night shift work experience and were working at least 3 overnight shifts per month. These work characteristics were chosen in an attempt to recruit participants who were regularly engaged in night shift work. Participants were recruited through paid Facebook advertisements, word of mouth and a registry of interested individuals from previous shift work studies conducted by the research group.

Participants expressed interest through completing an online screening questionnaire (Qualtrics, Utah, United States). The first author contacted eligible participants via phone, to explain the aims and procedures of the study. For those who agreed to participate, a briefing of the photo-taking activity was provided and the semi-structured interview was scheduled. Written informed consent was obtained prior to the interview, via email for those who were interviewed virtually and in hardcopy for those who were interviewed in-person.

### Data collection

This photovoice study included three data sources; (i) photos taken by the participants, (ii) demographics questionnaire and (iii) semi-structured interview.

#### Photo-taking activity

Participants were asked to take photos that conveyed, ‘*What are shift worker’s food choices and eating habits at work and outside of work?*’ Participants were asked to take an unlimited number of photos, on days when they were working night shift and also on rostered days-off, in the subsequent two to three weeks. Additional prompt questions and photo examples were provided to participants in a hand-out. Prompt questions illustrated to participants the times of the day where they may want to take photos, e.g. ‘Before I went to my night shift, I bought…’ and ‘During my day off I usually eat with…’. It was emphasised to participants that the photos would not be used as dietary assessments. Photos were taken on smart phones and sent to the researcher prior to the semi-structured interview.

#### Demographics questionnaire

Participants were asked to complete a demographics questionnaire via an online platform (Qualtrics, Utah, United States) prior to the semi-structured interview. This questionnaire gathered information on demographics (e.g. age, level of education and household composition) and shift work status (e.g. occupation, shift schedule and years in shift work).

#### Semi-structured interview

In order to understand the breadth of night shift workers’ experience with food and eating, the semi-structured interview was designed to explore three key areas using photos: (i) description of food choices and eating habits on night shifts and rostered days-off, (ii) factors influencing the aforementioned and (iii) perceptions and enablers to healthy eating, with a focus on workplace influence (interview guide – Table 1). The focus on workplace influence was chosen based on the research team’s knowledge of the literature. The key areas of the interview guide were ordered in a way to assist participants with reflecting on factors that influenced their eating habits, i.e. from describing what they ate to thinking about the reasons for their food choices. Interview questions were worded to allow participants ample opportunities to incorporate their photos in their responses. With such study design, interview responses and photos taken are equally valued as data sources, eliciting data richness and allowing for data triangulation. This was preferred over the SHOWED mnemonic method typically used in photovoice studies^(22)^, which relies on participants to take photos that encapsulate and convey experiences largely on their own.

**Table 1:** Interview guide for semi-structured interview

Section 1: Description of food choices and eating habits
Q1. Let’s start off by talking about what a typical day looks like. Using your photos, can you please tell me what a typical day looks like when you have to work night shift? Q2. Okay, now let’s take a focus on food. Again using your photos, tell me about your food choices and eating habits, on a day when you have to work night shift. Q3. Now let’s look at a typical day-off. Which of these photos look like your food choices and eating habits on a typical day off? Q4. Are there any differences or similarities in your food choices and eating habits between the two (a day when you are working night shift and a day off)? – Could you share with me why this is the case? – (for those who work other types of shifts) Did you take any photos on your day/ evening shift? Could you tell me about those?
Section 2: Factors that influence food choices and eating habits
Q5. (Refer back to photos) What influenced your decision to choose this meal/snack? Q6. Comparing a day when you are working night shift to a day when you have a day-off. Are there any similarities or differences in the factors that influence your food choices and eating habits?
Section 3: Perceptions and enablers of healthy eating
With your experience as a shift worker, I would like to know your opinion on ways that can help shift workers practice healthy eating. Q7. But “healthy eating” can mean a whole range of different things to us. Using your photos, can you please show me what it means to you? Q8. Is this meaning different when you are at work compared with not at work? Can you explain this to me using your photos? Q9. Can you please share with me, what are some of the enablers that help you/ will help you make these choices or maintain these habits? Q10. (PICK PHOTOS that are relating to workplace – discussed or new) All of these photos here relates to your workplace. Several workplaces that employ shift workers have said that they would like to support healthy eating. – Do you have any suggestions for them, on how to do this?

During the interview, all photos that the participant took were displayed to them either in hardcopy or on a PowerPoint Presentation. Participants were asked to select photos and use them in their descriptions of experiences and reflections. No guidance was provided regarding which and how many photos to use.

Using a semi-structured interview format, the main questions asked were based on the three key areas, with prompting questions based on participants’ photos, their responses in the interview and the demographics questionnaire. The interviews took place in-person (in a meeting room at our research facility) or virtually (via Zoom (Zoom Video Communications, California, United States)). All interviews were conducted by the first author, whom was introduced to the participants as a researcher rather than a dietitian, to prevent participants from feeling judged based on their dietary habits. From their dietetic training, the first author was aware of factors that typically influence individuals’ eating habits, which led her to choose particular prompting questions during the interview. This rationale, as well as assumptions and tentative codes, were documented by the first author in field notes, taken prior to and after each interview as part of critical reflexivity practice. All interviews were audio-recorded and subsequently transcribed verbatim.

### Data analysis

The NVivo software (QSR International, Version Pro 12) was used to manage and support data analysis. Interview transcripts were uploaded to the software, with photos linked to the discussed sections of dialogue. The thematic data analysis approach was taken, via an iterative inductive coding process. Prior to coding each transcript, the first author reviewed the post-interview field notes to familiarise themselves with the context of the interview and any tentative codes noted. The first author and last author cross-checked two interviews to develop the initial codebook. The first author then coded remaining interviews, meeting with the last author to refine the codebook after every three interviews. The codes were grouped into categories and then allocated into themes and subthemes, which were refined and verified by the two authors. As dietitians, the two authors acknowledged that individuals’ food choices and eating habits are influenced by interconnecting factors; a mind map was therefore developed to illustrate the complex network of associations. Four themes with corresponding subthemes, illustrative quotes and photos; and the mind map are presented in the results. All participants were provided with a pseudonym to preserve confidentiality.

## Results

During the recruitment period, thirty-nine individuals expressed interest in the study. Ten participants completed the study, with twenty-nine others either ineligible or did not respond to invitation to participate. Majority of participants were engaged in the healthcare, emergency services or security sector (Table 2). All participants were located in the state of Victoria, except one who was located in the state of Queensland. Number of photos taken by participants ranged from 4 to 29, mainly depicting food items consumed during shifts or on rostered days-off, followed by their eating environment. Six interviews were conducted virtually (Zoom) and four were conducted in-person, ranging from 20 to 82 min in length.

**Table 2:** Demographics characteristics of participants (*n* 10)

Demographics	Frequency^*^
Age (years), range	19–66
Gender	
Female	4
Male	6
Occupational fields	
Healthcare	2
Emergency services and support	3
Security	3
Hospitality and food service	1
Avionic maintenance	1
Form of shift work	
Permanent night shift	4
Rotating shifts^†^	6
Years in shift work, range	2 – 25
Household composition	
Living alone	3
Living with others	7

* Frequency presented unless otherwise stated.

† Rotating shifts include a combination of day and night shifts.

**Fig. 1:**
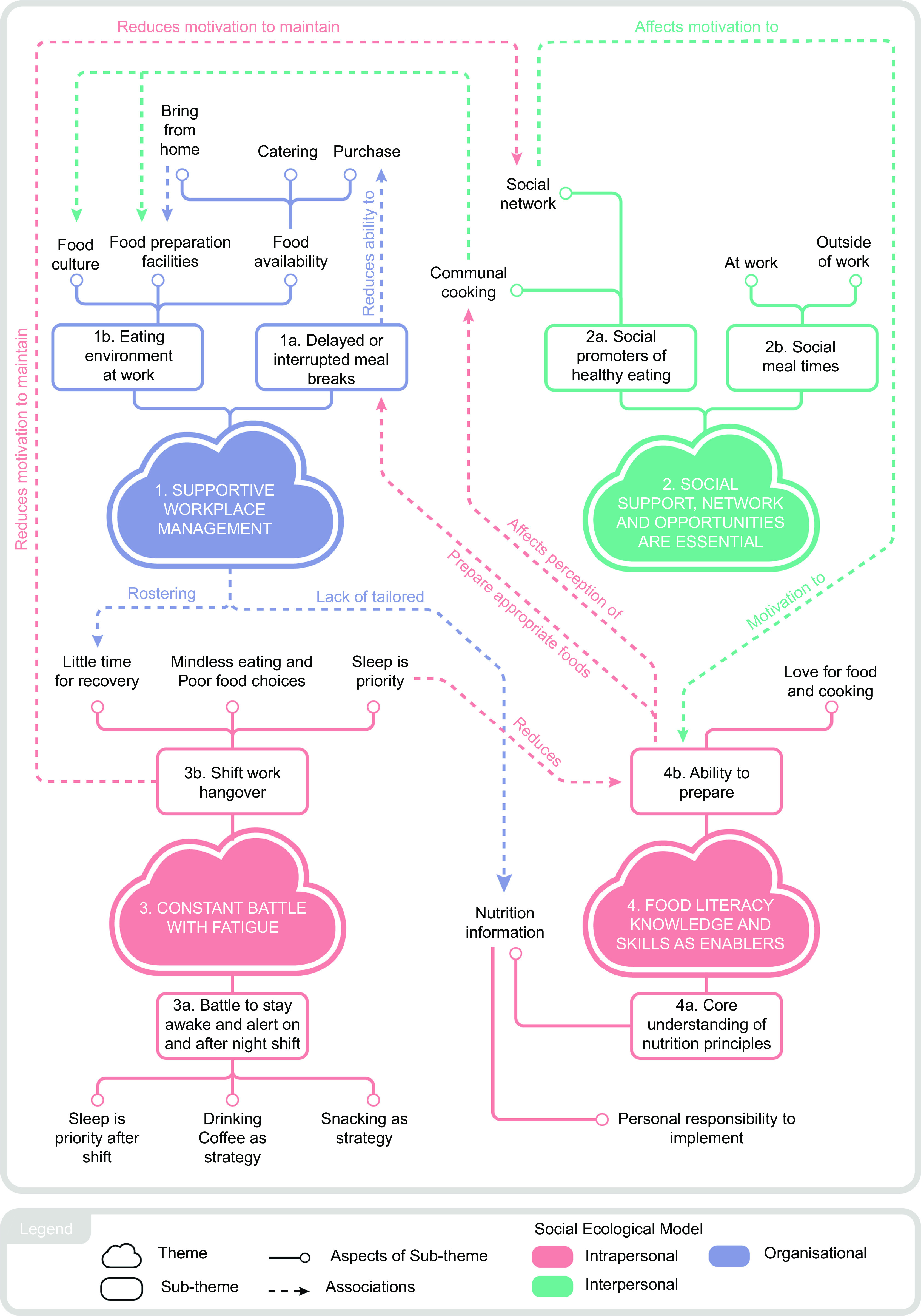
Mind map describing themes and subthemes that influence night shift workers’ food choices and eating habits. Our participants described that their eating habits are shaped by a combination of interconnecting factors, which are illustrated by the ‘associations’ in the figure. Themes and subthemes have been mapped onto the Social Ecological Model^(23)^, which categorises influences of behaviour into intrapersonal, interpersonal or organisational levels.

Data analysis yielded four interactive themes, each with two subthemes, describing factors that influence night shift workers’ food choices and eating habits: 1) supportive workplace management contributes to enabling workplace food and eating environments; 2) social support, network and opportunities are essential to shift workers; 3) constant battle with fatigue and 4) food literacy knowledge and skills as enablers. Themes and subthemes developed are illustrated in a mind map (Fig. 1); defining aspects are also provided under each subtheme, explaining the ideas that each subtheme encapsulates. Our data show that night shift workers’ eating habits are not shaped by a single factor, rather, a combination of factors that may be positioned under different themes; this interconnection is reflected by the ‘associations’ in Fig. 1. Themes and subthemes are also mapped onto the Social Ecological Model^(23)^ in Fig. 1, which categorises influences of behaviour into intrapersonal, interpersonal and organisational levels, and will be referred to in the discussion of findings.

### Theme 1: supportive workplace management contributes to enabling workplace food and eating environments

Participants perceived aspects of the meal break structure and workplace food environment as impractical, and that it reflected management’s lack of consideration for their health and wellbeing. This was particularly evident for permanent night shift workers, who indicated they ‘*don’t really have a voice*’, as staff meetings and health initiatives were often scheduled at times convenient for day-time staff.

#### Subtheme 1a: delayed or interrupted meal breaks

**Fig. 2:**
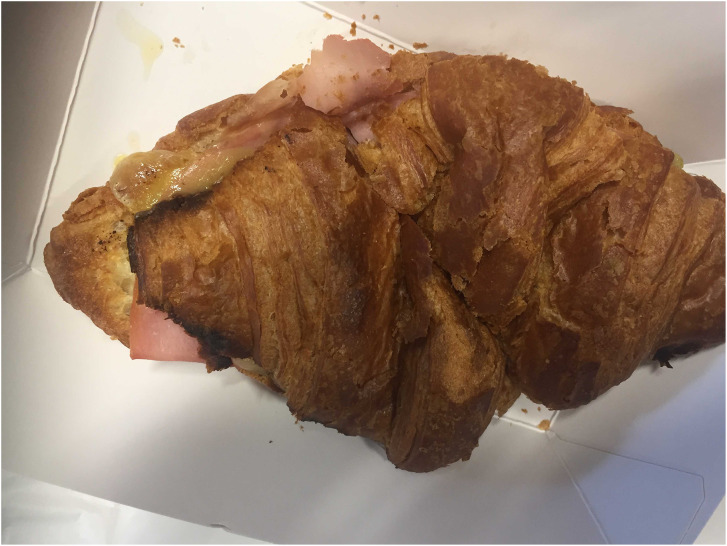
‘*Front loading*’ food and caffeine was common, if meal breaks were expected to be delayed. (Nathan)

Most participants did not have designated break times, during both night and day shifts. Fulfilling work responsibilities was prioritised; therefore, meal breaks were often delayed, missed or interrupted. Many would ‘*front load*’ meals and caffeine, as Nathan reflected on his choice of a croissant (Fig. 2) during his afternoon shift, *‘In that situation you’re not like really hungry coz you’ve already eaten [lunch]. But you’re worried that you will get tied up for the rest of your shift’.* Front loading was not limited to night shifts and was dependent on the anticipated workload. Delays in meal breaks during night shifts particularly affected those who relied on purchasing foods, limiting their options to fast foods only.

#### Subtheme 1b: eating environment at work

Availability of food options and food preparation facilities varied depending on the workplace physical environment and culture. For participants who relied on purchasing food while at work, unhealthy food options were more common during night shifts compared with day shifts, due to reduced availability of food outlets. Belinda described that on a day shift, *‘Your options are better, because if you don’t bring your food, you can just go and get something that’s not too bad?’* In comparison, all cafés near her workplace are closed during the night, therefore, *‘By the time I could go and get something to eat, it was KFC’.*

Some participants were not permitted to leave their worksite during shift, therefore relied on food brought from home. This required a level of food literacy from the individual or supportive family members to plan and prepare food, as well as the availability of food storage and preparation facilities in the workplace. As described by Cassey, who could prepare convenience foods in her current workplace equipped with facilities. In contrast to her previous workplaces*, ‘where my [her] break room has been a [street] alleyway’.* Participants perceived the lack of food preparation facilities indicative that management *‘don’t seem to understand or care about healthy eating’.*

Catering was provided by some workplaces, but options were not necessarily healthy or desired. The culture of sharing discretionary foods was common across multiple workplaces and especially prominent during night shifts, when workload is reduced or a mood boost was required.

### Theme 2: social support, network and opportunities are essential to shift workers

Being on night shifts was described as ‘*isolating*’ by both permanent and rotating shift workers. Lingering fatigue associated with night shift reduced participants’ motivation to engage in social activities, *‘You’re so mentally and emotionally and like physically drained, you don’t have any time for it. You just don’t have the effort.’* (Nellie) This had a negative impact on workers’ eating habits, in that they were less likely to engage in healthy eating practices in the absence of social support. Support from family and significant others were crucial in order to cope with the disruptions of night shift work.

#### Subtheme 2a: social meal time

Outside of work, meal times were opportunities for socialisation to maintain relationships with family and friends. These opportunities were invaluable due to limited socialisation during night shifts. Having company at meal times also prevented participants from skipping meals or choosing convenience foods.

**Fig. 3:**
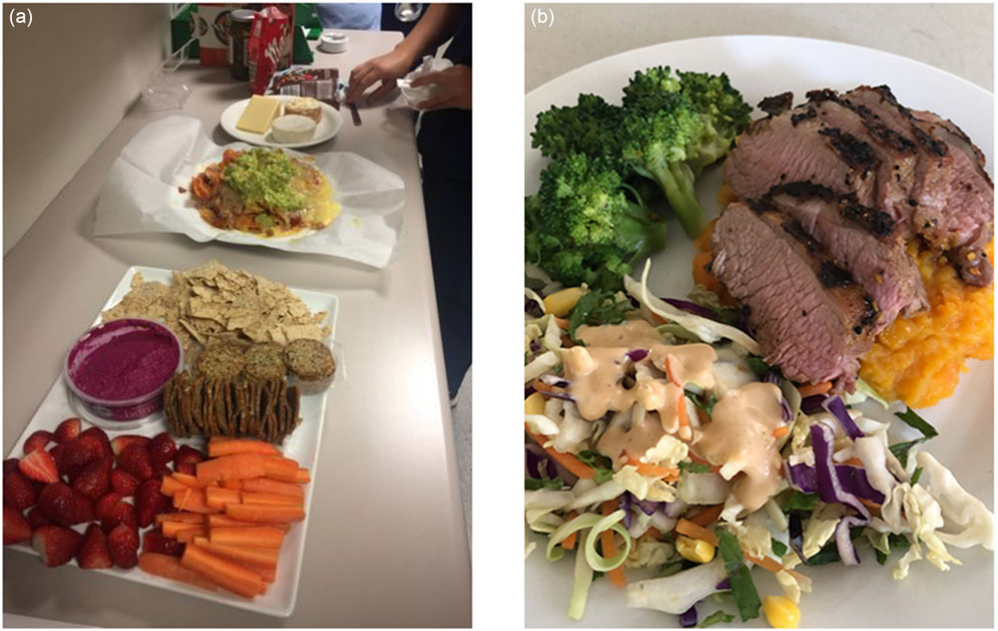
(a) Eating is a social activity during night shifts. In the hospital setting, staff would set up grazing platters at the main nurses’ station, where they would gather during their breaks (Nellie). (b) Having social network was associated with participants’ motivation to prepare food. Susan prepared dinner for her family and set aside a serving to be brought to work.

Meal breaks during night shifts also became opportunities for social interaction. As Samuel reflected, *‘We go upstairs and we have coffee when we have chances. And we try to do it every night, it’s just more of a social thing as well’.* Nellie described the contrasting food environment between day and night shifts in a hospital setting. During night shifts, a grazing table (Fig. 3(a)) would often be set up at the main nurse’s station, if those working were friendly with each other. In contrast, official breaks were undertaken in the staff room during day shifts. Nellie reflected, *‘At night there’s less of you [staff], and so you kinda (sic) gravitate towards each other. Coz (sic) a lot of the time the only social interaction on night duty is the other staff’.*

#### Subtheme 2b: social promoters of healthy eating

Living with others was closely associated with participants’ motivation to prepare food. Belinda reflected that it is difficult to be organised with meal preparation because she is single. She noticed that mothers at her workplace would often bring plated meals to work, which were prepared because they had to cook for their children. This was evident in one participant Susan, who prepared dinner (Fig. 3(b)) for her family and set aside a serving to be brought to her night shift.

Staff-initiated communal cooking was reported in some workplaces, but relied on the availability of food preparation facilities in the workplace. Otherwise, it was dependent on particular motivated individuals, who would bring prepared dishes or cooking appliances to work.

### Theme 3: constant battle with fatigue

Participants expressed experiencing a constant state of fatigue caused by shift work, in particular night shift work. Food was used as a means to fight fatigue during night shifts. However, the lingering impact of fatigue from night shift work affected eating habits negatively.

#### Subtheme 3a: battle to stay awake and alert on night shift

During night shifts, snacking was used as a measure to stay awake and focussed. As Nellie described, *‘You’re not even hungry, but you just need that something to do to keep you awake… That’s why you can’t skip chips…’*

Coffee consumption was commonly used to stay awake. Participants who drank coffee regularly ensured that they had a sufficient caffeine intake during night shifts, as they were concerned about falling asleep during work and on their commute home after a night shift.

**Fig. 4:**
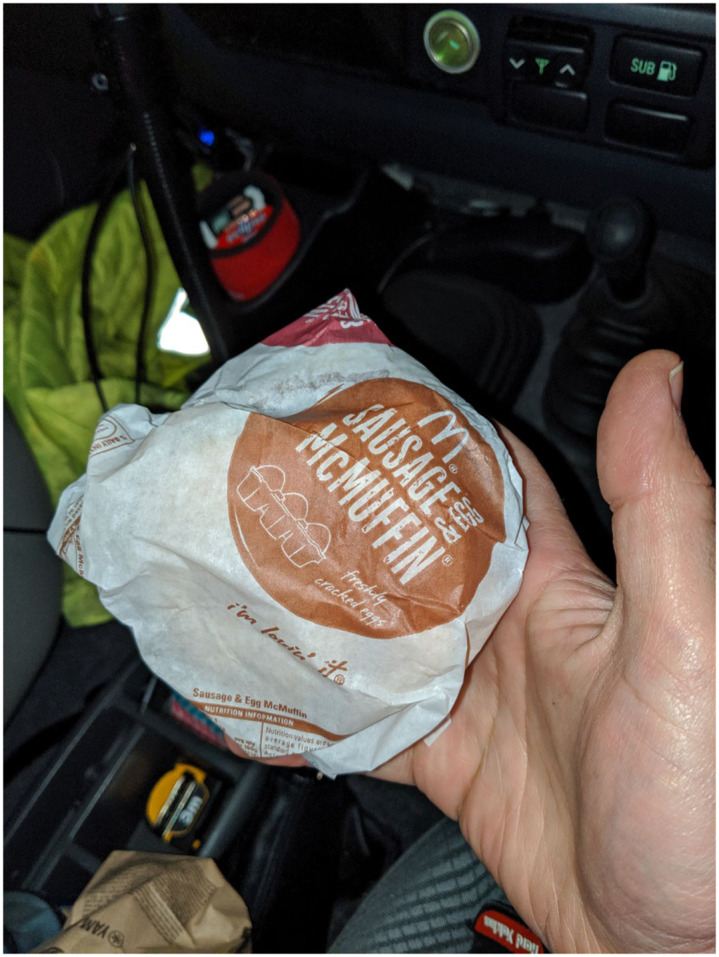
Samuel chose a convenient food option, consumed during the commute home after a night shift, so that he could get to bed as soon as possible.

Participants expressed being exhausted after night shifts and would often eat something that was quick and convenient (for example, Fig. 4) or skip meals, so they could get to bed as soon as possible and maximise their sleep opportunity.

#### Subtheme 3b: shift work hangover

Night shifts led to fatigue that lingered in between and after consecutive night shifts, creating the experience of a ‘hangover’, which had an impact on food choices and preparation. During a block of night shifts, getting enough sleep in preparation for the next shift was considered priority over eating. As Samuel reflected, *‘I’m not as interested in healthy eating on my days-on, as I am on my days-off… You’re just more focussed on sleeping, rather than going to the shops and getting food… When I’m at work, it’s really just work and sleep.’*

Participants reported having little time or motivation in between night shifts for food preparation tasks such as grocery shopping or cooking. Therefore, if they were not organised prior to a block of night shifts, they would resort to convenience foods. Even if they had groceries prepared, the decision of whether they would cook before going to their night shift was largely dependent on whether there was time to get enough sleep.

Fatigue accumulated during a block of night shifts would often extend into participants’ rostered days-off. Participants reported requiring the first couple of days-off to recover; their eating habits were subsequently affected. Evidenced by Nellie’s experience, *‘My first few days after a lot of night duties, my body clock is still out-of-whack… Even during my days off after night duties, it takes a little while for me to get back into an eating pattern.’* This lingering fatigue led to reduced motivation to cook despite the best intentions. As Samuel described, he attempted to do grocery shopping on his first day-off and was prepared to make dinner. However, he ‘*hit a wall*’ and resorted to food delivery. The effect of ‘shift work hangover’ was more apparent in rotating shift workers compared with permanent night workers. This was likely due to constant changes in shift types and structure of rosters, which allowed little time for recovery in between and after a block of shifts.

### Theme 4: food literacy knowledge and skills as enablers

Participants had an adequate understanding of core nutrition principles. However, this did not reduce the difficulties they faced in maintaining healthy eating habits, with numerous external factors described in Themes 1, 2 and 3 acting as barriers. The shift work lifestyle had significant impacts on participants’ ability to prepare food; however, the enjoyment for food and cooking seemed to act as a motivator for some.

#### Subtheme 4a: core understanding of nutrition principles

**Fig. 5:**
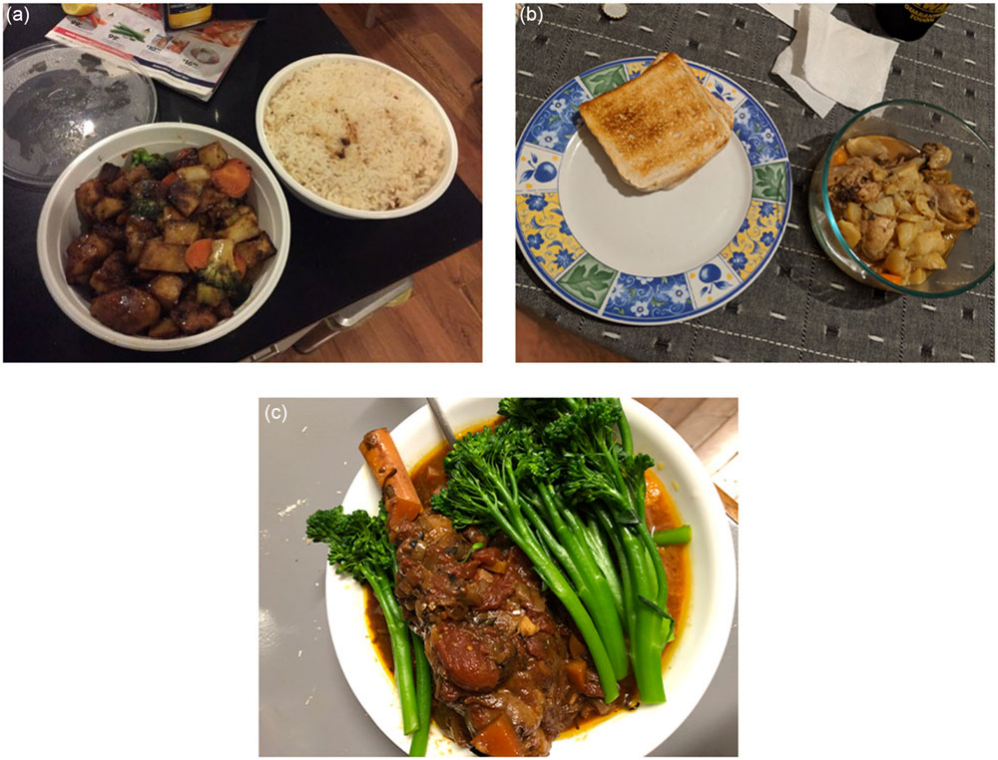
(a) ‘Meal prep’ done prior to night shifts, using grocery delivery service. Nellie added extra vegetables into the dish, quoting the importance of a ‘balanced’ meal. (b) Samuel’s perception of a ‘healthy meal’, with vegetables, meat and grain foods. (c) Dinner that Susan prepared using a slow cooker. Those who enjoyed cooking had knowledge of ‘shortcuts’ that could be used to reduce food preparation time.

All participants could describe the principles of healthy eating. When asked about their interpretation of healthy eating, majority identified the importance of ‘*balanced*’ meals or diet (Fig. 5(a) and (b)), with a focus on vegetables, but also including meat and grain foods.

Participants had their own way of planning meal times around their shift schedule, based on hunger, workload and/or break times. Some workplaces attempted to meet occupational health and safety requirements by providing nutrition information to employees, for example, via regular online training modules. However, this was described as *‘dry training’* that was *‘forced upon’* them and were not tailored to their needs as shift workers. Participants voiced that the management’s inability to provide tailored nutrition information accentuated the employer’s indifference to their health and well-being.

Maintaining or practising healthy eating was described as a ‘*battle*’ by participants. Through the narration of their experiences, participants seemed to be aware of how their eating habits were affected by external factors, such as short or delayed meal breaks, the workplace food environment and culture. Despite recognising these factors, when asked explicitly about factors that help them practise healthy eating, participants perceived this as largely their responsibility. They stated needing to be *‘disciplined’, ‘more organised’* and be more *‘conscious’* about their food choices.

#### Subtheme 4b: having the ability to prepare is crucial

Participants reported having little time for food preparation tasks; mainly attributing to the experience of shift work hangover, and often relied on convenient food options. However, perceptions of ‘*convenient*’ food options varied amongst participants and was related to their enjoyment of food and cooking. Those who ‘*loved being in the kitchen*’ were more likely to view having a proper meal as a ‘*necessity*’. They would, therefore, know how to use cooking appliances and ingredients to create ‘*shortcuts*’ and reduce food preparation time. As shown by Susan, *‘That* [Fig. 5(c)] *would be done in a slow cooker at the start of the day, all the ingredients thrown in and then served when I get home for dinner’.* Participants who expressed an enjoyment for food and cooking would also tend to ‘*meal prep*’, cooking a batch of meals on their days-off, in preparation for their night shifts (Fig. 5(a)).

## Discussion

This qualitative study is the first to use the photovoice enquiry method to explore the food experiences of Australian night shift workers from different occupations. Four main themes emerged from the data that describe key influences of workers’ food choices and eating habits both within and outside of the workplace, the latter of which is a novel aspect of the study. Our findings indicate that night shift workers’ food choices and eating habits are influenced by a complex interplay between multiple individual, social and environmental factors. Workplace management played a crucial role in supporting healthy eating practices within the workplace, through ensuring food availability, providing food preparation facilities and appropriate meal break scheduling. In addition, their arrangement of shift rosters had an indirect impact on workers’ eating habits outside of work. Shift rosters provided little time for recovery between shifts, leading to the experience of shift work hangover, which was evident in many participants’ stories. This affected workers’ abilities and motivations to engage in healthy eating practices both on work-days and days-off, despite their best intentions. In this context, the presence of a supportive social network and being equipped with adequate food literacy skills were essential, to reduce the perceived efforts required to engage in healthy eating.

The complex interplay of individual and environmental factors that shaped participants’ food choices and eating habits is fittingly described by the Social Ecological Model (SE Model)^(23,24)^. This model explains human behaviour is influenced by the cumulative impact of environmental factors at the interpersonal, organisational, community and public policy level^(24)^. However, the same environment may affect each individual’s behaviour differently, dependent on intrapersonal factors such as personality and perceptions of environmental controllability^(24)^.

Workplace management is able to make significant contributions to workers’ health, through the provision of healthy workplace setting. Described by the World Health Organisation, a healthy workplace setting can be created through modifications in the workplace environment and organisational structures^(25)^. Such environmental enablers have the potential to promote sustainable behaviour change, as they do not require continuous voluntary efforts from individuals^(24,26)^. Despite limited evidence from the shift work literature specifically, health promotion programs conducted in manufacturing companies, which typically employ shift workers, have indicated the effectiveness of workplace environmental modifications on improving workers’ dietary intake. The Well-Works Study implemented strategies targeting multiple levels of the SE Model, including health education programs (intrapersonal level), changes to food items available in canteens (organisational) and food catering policies (local policy)^(27)^. The study included 24 manufacturing worksites (2658 participants) in the USA and reported an increase in fruit and vegetable intake and a reduction in fat intake at the end of the 2-year intervention. These findings were supported by the Food Choice at Work trial conducted in manufacturing worksites in Ireland^(28)^. This 7-month trial involved four worksites including a Control site (no intervention), an Education site (individual and group nutrition education), an Environmental site (menu modifications and strategic position of canteen items) and a Combined site (Education and Environmental). Whilst a reduction in participants’ BMI, dietary saturated fat and salt intake were observed in the Combined intervention, the Education and Environmental interventions alone had minimal effects. Based on our study’s findings, a healthy workplace setting for night shift workers could include food storage and preparation facilities that allow workers to bring food from home and ensure that healthy food options are also available for purchase during the night.

From the workers’ perspective, the absence of a healthy workplace setting also reflects the management personnels’ indifference on their health. Our participants expressed that their workplace management did not acknowledge barriers to healthy eating and failed to provide supportive measures accordingly. Consequently, they may feel undervalued, leading to low self-efficacy and the adoption of a ‘why bother’ attitude towards making positive lifestyle changes^(29)^. This highlights that workplace management not only affect workers’ abilities to practise healthy eating through the provision of supportive environments, but their attitudes also have an indirect influence on workers’ perceived need and capacity to adopt dietary changes. Our participants indicated that the provision of nutrition education through online modules was not considered helpful and did not reflect the management teams’ interests on their health. The general workplace literature suggests several interventions that are effective in promoting healthy eating habits such as workplace health promotion campaigns, provision of dietary counselling and financial incentives^(30)^ and may convey the management teams’ considerations on their employees’ well-being^(31)^. Some of these interventions may be tested for effectiveness in the night shift worker population; however, strategies need to be implemented to ensure access for night shift workers.

Fatigue has been repeatedly reported as a key detriment of night shift work^(29,32,33)^. Participants highlighted that tiredness lingered beyond their shifts and impacted on their abilities to make healthy food choices even on their rostered days-off. A cross-sectional study including 118 Australian shift workers showed that increased levels of fatigue are associated with increased daily fat intake (as percentage energy)^(15)^. Moreover, a simulated night shift study reported that sleep restriction (5·5 h per night) increased snack consumption, in particular sweet snacks, compared with regular sleep duration of 8·5 h per night^(34)^. These unhealthy eating habits are likely to be maintained in night shift workers’ daily lives, as they find it difficult to recover from fatigue caused by night shift work. As such, health promotion strategies should also aim to improve night shift workers’ eating habits beyond that observed within the workplace. This can take the form of nutrition education focusing on building food literacy, specifically the ‘ability to prepare food’^(35)^. Education on time-efficient skills to prepare healthy food would be valuable, so that workers can maintain their prioritisation on sleep and rest whilst off-work.

It is evident that night shift workers’ food choices and eating habits are shaped by interacting factors situated in various levels of the SE Model. Hence, the most effective health promotion programs are those that utilise a combination of strategies, targeting multiple levels of influences^(24,27,28)^. In addition to strategies described above, constant fatigue (intrapersonal factor) can also be effectively addressed through health and safety regulations on appropriate rostering, mandated at the workplace organisation and/or public policy level. Evidenced by our findings, inappropriate rostering is an issue present within multiple shift work industries, allowing little time for workers to recover in between and after night shifts. A recent expert discussion paper suggested that night shift rosters should have no more than three consecutive night shifts and intervals between two shifts should be at least 11 h, in order to prevent fatigue-related injuries^(36)^. If implemented, such policy is likely to have a flow-on effect and enable healthy eating habits. Workplace management can also consider harnessing existing workplace cultures such as communal cooking. Healthy eating habits could be promoted through this avenue, by creating supportive environments such as the provision of appropriate food preparation facilities, ingredients and recipes. A secondary effect of this is the cultivation of social networks between colleagues, an interpersonal influence of well-being that is affected by night shift work. Evidently, support from workplace management is essential in the implementation of health promotion strategies for this population. However, engagement with industry is difficult to establish, with majority of employers expressing a lack of interest in healthcare interventions, due to the absence of direct benefits^(37)^. Health promotion strategies for this population should therefore include the evaluation of work-related outcomes. Studies have shown that workplace nutrition and physical activity interventions are able to reduce absenteeism and increase work productivity^(38–40)^, which directly contribute to profits of businesses.

### Strengths and limitations

Method triangulation was achieved through the combined use of multiple data collection methods, including photo-taking, semi-structured interviews, field notes and demographic questionnaires. This generated complementary data sources, allowing researchers to verify research findings and thereby increasing their validity^(41)^. During the interviews, the incorporation of photos prompted reflections, memory and description of experiences that are indirectly related to the photos^(42)^. Moreover, participants reported that the photo-taking activity itself helped them notice and acknowledge their daily eating habits^(43)^. The exploration of night shift workers’ experiences outside of work was a novel component of this study’s research question. Although participants were not able to clearly distinguish their food experiences on days-off from days at work, they often referred to the burdens of night shift work when describing their eating habits on days-off.

### Conclusion

This study’s findings confirmed that night shift workers’ food choices and eating habits are shaped by a complex interplay of individual and environmental factors. Workplace management plays a crucial role in creating physical environments that support healthy eating habits. Moreover, workplace and public policies regulating meal break and roster structures exacerbate intrapersonal barriers to healthy eating, such as constant fatigue and reduced motivation for food preparation. These factors not only hindered workers’ abilities to make healthy food choices at work but also continued to affect their eating habits on rostered days-off. As such, health promotion strategies should consider targeting dietary behaviours within and external to the workplace setting. More importantly, health promotion for this population requires support from workplace management and should have a multi-strategy approach, which targets multiple levels of the Social Ecological Model, ranging from interventions targeted at the individual, to workplace-settings based approaches and organisation and/or public policy reforms.

## Supplementary material

For supplementary material accompanying this paper visit https://doi.org/10.1017/S1368980023001519. click here to view supplementary material
